# War Archives: How Some Primates Fought Off Ancient Viruses

**DOI:** 10.1371/journal.pbio.1001285

**Published:** 2012-03-13

**Authors:** Caitlin Sedwick

**Affiliations:** Freelance Science Writer, San Diego, California, United States of America

**Figure pbio-1001285-g001:**
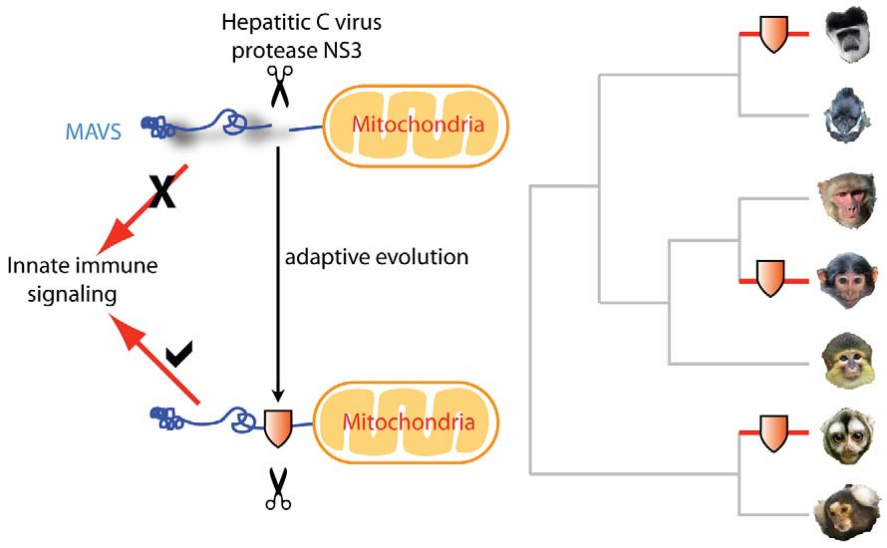
Hepatitis C virus cleaves antiviral factor MAVS to evade host detection (left diagram, top). Adaptive changes (red shields) close to the cleavage site provide protection from this antagonism and have occurred in multiple primate species via convergent evolution (right diagram).

[Fig pbio-1001285-g001]For eons, viruses and their host organisms have been locked in an evolutionary arms race, with hosts—sometimes under threat of death—continually perfecting adaptive countermeasures to thwart viral threats. Such adaptations often come about via mutation in single amino acids of host proteins. The mutations become fixed in the host population as resistant individuals and their offspring outperform susceptible individuals. When this happens, the mutation is said to have undergone positive selection. In this issue of *PLoS Biology*, Maulik Patel, Harmit Malik, and colleagues explain how selective pressures from ancient viruses related to hepatitis C virus (HCV) sometimes forced adaptations in a protein, MAVS, that is critical for primate antiviral responses.

When cells detect the presence of RNA viruses such as HCV, MAVS (short for Mitochondrial antiviral signaling) acts to kick-start the production of potent antiviral factors that can stop viral replication. Unfortunately for humans (the only primates naturally infected by HCV), the virus can trump this defense using the viral protein NS3, which chops up and inactivates MAVS. This is part of the reason HCV is such a nasty virus for humans, causing liver disease in many of the approximately 200 million infected people worldwide.

For insight into why human MAVS is so vulnerable to HCV, the researchers examined MAVS proteins in other primates. Statistical analyses of MAVS amino acid sequences from 21 primate species, including humans, indicated that MAVS has undergone positive selection in several primate species at some point in the past. This suggests many primates have probably faced infection by viruses that attack MAVS.

What kinds of changes did these viruses provoke in MAVS? Might some of these changes be aimed at protecting against a viral protein similar to HCV NS3? To address these questions, the authors first showed that all the primate MAVS could support production of antiviral factors. Then they examined whether any could do so when HCV NS3 is present. They found that three primate species (rhesus macaques, spider monkeys, and dusky titis) possess MAVS that is very resistant to suppression by HCV NS3. These “resistant” MAVS each exhibit a mutation substituting a different amino acid for the valine that normally appears at position 506 in MAVS of all the other primates tested (including humans). The researchers showed that mutations at position 506 cause resistance because when they changed the mutations back to valine, resistance disappeared.

Surprisingly, none of the three primates with resistant MAVS are closely related to each other, so they must each have independently evolved their protective mutations at position 506. Nonetheless, these mutations all likely work in the same way: by stopping NS3 from binding tightly to MAVS and thereby preventing the inactivation of MAVS. That's why NS3-resistant MAVS, like that found in rhesus macaques, or in human MAVS where “resistant” mutations have been experimentally introduced, controls HCV replication in cell culture so much better than does the normal human MAVS protein.

Together, these data support the idea that mutations at position 506 happened in primates that were under pressure from viruses that, like HCV, could cleave MAVS. These mutations weren't driven by HCV (because in nature, HCV only infects humans), but instead, something similar. In fact, there are a few viruses distantly related to HCV that infect wild primates. These viruses possess their own NS3-like proteins, which Patel and colleagues showed could interfere with the ability of normal human MAVS to stimulate antiviral factor production. Therefore, the ability to antagonize MAVS is likely a key feature of this viral family.

Patel and colleagues point out that today, no HCV-like viruses are found in primate species that have NS3-resistant MAVS, so we don't actually know much about the viruses that drove positive selection at position 506. Nonetheless, we can infer that they possessed a protein similar to NS3. We can also deduce that they also last plagued their hosts long ago: rhesus macaques share their protective mutation with other macaque species but not with their next closest primate relatives (baboons). Thus, the mutation probably appeared around the time the two primates split off from each other, 5–8 million years ago.

Although humans still struggle with HCV, the evidence presented in this paper suggests that some primates have won their evolutionary arms race. Sometimes, such vanquished viruses leave behind quiescent “fossils” integrated into their hosts' DNA. But no such fossils have yet been found and these ancient viruses may now be extinct. In that case, the only way to learn anything about them is to study the armaments (mutations) they inspired in their host's genomes, using methods like those employed by Patel et al.

**Patel MR, Loo Y-M, Horner SM, Gale M Jr, Malik HS (2012) Convergent Evolution of Escape from Hepaciviral Antagonism in Primates. doi:10.1371/journal.pbio.1001282**

